# Interpreting the potential of biogenic TiO_2_ nanoparticles on enhancing soybean resilience to salinity via maintaining ion homeostasis and minimizing malondialdehyde

**DOI:** 10.1038/s41598-025-94421-3

**Published:** 2025-04-15

**Authors:** Reda E. Abdelhameed, Hanan Abdalla, Hegazy S. Hegazy, Marwa H. Adarosy

**Affiliations:** Botany and Microbiology Department, Faculty of Science, Zagazig University, Zagazig, 44519 Egypt

**Keywords:** Salinity, Nanoparticles, Water content, Lipid peroxidation, Phenolics, Carbohydrates, Minerals, Biological techniques, Physiology, Plant sciences

## Abstract

The use of nanoparticles has emerged as a popular amendment and promising approach to enhance plant resilience to environmental stressors, including salinity. Salinity stress is a critical issue in global agriculture, requiring strategies such as salt-tolerant crop varieties, soil amendments, and nanotechnology-based solutions to mitigate its effects. Therefore, this paper explores the role of plant-based titanium dioxide nanoparticles (nTiO_2_) in mitigating the effects of salinity stress on soybean phenotypic variation, water content, non-enzymatic antioxidants, malondialdehyde (MDA) and mineral contents. Both 0 and 30 ppm nTiO_2_ treatments were applied to the soybean plants, along with six salt concentrations (0, 25, 50, 100, 150, and 200 mM NaCl) and the combined effect of nTiO_2_ and salinity. Salinity decreased water content, chlorophyll and carotenoids which results in a significant decrement in the total fresh and dry weights. Treatment of control and NaCl treated plants by nTiO_2_ showed improvements in the vegetative growth of soybean plants by increasing its chlorophyll, water content and carbohydrates. Additionally, nTiO_2_ application boosted the accumulation of non-enzymatic antioxidants, contributing to reduced oxidative damage (less MDA). Notably, it also mitigated Na^+^ accumulation while promoting K^+^ and Mg^++^ uptake in both leaves and roots, essential for maintaining ion homeostasis and metabolic function. These results suggest that nTiO_2_ has the potential to improve salinity tolerance in soybean by maintaining proper ion balance and reducing MDA level, offering a promising strategy for crop management in saline-prone areas.

## Introduction

Nanobiotechnology is an effective innovation that deals with materials that are nanometres in size in a variety of scientific domains. These materials are widely used in many parts of agriculture, including plant nourishment, plant protection and as nano pesticides^1,2^. Due to the growing need for nontoxic chemicals, antiviral, antibacterial, diagnostic, anticancer, targeted drug delivery, environmentally friendly solvents, and renewable resources, biosynthesis of nanoparticles (NPs) has received a lot of attention in recent years^3,4^. The green synthesis method is environmentally benign because it uses bacteria, fungi, and plant extracts (leaves, flowers, seeds, and peels) to create NPs rather than a lot of chemicals^5^. Among the various types of NPs, titanium dioxide nanoparticles (nTiO_2_) have variety of uses due to their special qualities, which set them apart from many other NPs. These qualities include their tiny size, large surface area, non-toxicity, biocompatibility and their stability to various environmental circumstances. They also support agricultural plant growth, increase yield, and are utilized in food and cosmetics^6^. The nTiO_2_ have effects on plants, ranging from negative to neutral to favorable. Most importantly, when the appropriate concentrations are chosen, these NPs are used in ameliorating the harmful effects of plants under different stress conditions. It claimed that plants might better withstand a variety of environmental challenges when exposed to nTiO_2_, since it modulates a number of physio-biochemical mechanisms^7^.

One of these environmental challenges that face crops globally, leading to yearly crop losses, is salinity. Salinity stress, one significant environmental element, poses challenges to plant growth and development. Saline soils are brought about by a high accumulation of soluble salt particles of sodium (Na^+^) and chloride (Cl^−^) being the most harmful stress to plants^8^. High salt concentrations in the soil make it harder for the roots to absorb water, which causes water shortages in the plant tissues. By raising the levels of Na^+^ and Cl^−^ in plant cells, salinity stress alters a variety of plant morphological, physiological, epigenetic, and genetic traits. As a result, plants may encounter stunted growth, reduction in biomass accumulation and reduced development^9^. Different effects are being imposed by soil salinity such as ion toxicity, lack of necessary elements (N, P, K, Ca, Fe, Zn), osmotic stress etc. and delays the water uptake of plants from soil^10^. Stress brought on by salinity causes an excess of reactive oxygen species (ROS), which damage cellular membranes and components oxidatively and, in extreme saline situations, kill cells and plants. Through the antioxidant defense mechanism, plants prevent oxidative damage caused by salt by removing ROS and controlling their formation.

Plants can partially mitigate salinity disorder and restore the cell to its initial form through a variety of processes; nevertheless, if the salt dosage is excessive, the plants might not be able to respond appropriately and may perish from salt stress. The application of NPs has gained popularity recently as a way to reduce salinity stress and has recorded very brilliant results^9,11^. When NPs are applied as a foliar spray or seed priming agent, they activate germination enzymes, maintain ROS homeostasis, promote the synthesis of suitable solutes, and stimulate antioxidant defense systems, all of which improve crop quality and production^12^. One advantage of foliar application is that it uses fewer NPs, which results in less soil contamination and makes it more sustainable. The beneficial effects of nanotechnology on plants under salinity stress have been validated by several researchers^13,14^. Most convincingly, Mustafa et al.,^15^ demonstrated that the exogenous application of green nTiO_2_ increased the germination, physiochemical, and yield indices of wheat plants under salinity stress. Also, nTiO_2_ application enhanced the biochemical attributes: free amino acids, soluble sugar content, proline (Pro) content, and antioxidants of plants under different salinity levels^16^.

One of the principal crops affected by different abiotic stresses is soybean (*Glycine max* L.) which is one of the main oil crops that has several applications and is becoming more and more appreciated globally. It is an economically significant crop and a vital source of protein and oil worldwide^17^. Due to their recognized partial sensitivity to salt, a salt-sensitive legume, soybeans can lose up to 40% of their production, depending on the salinity level. Excessive salt in the soybean growing medium has a detrimental effect on the nodulation process, growth, and seed quality and quantity^18^. Salt stress negatively impacts a number of metabolic pathways, including protein synthesis, cytosolic and mitochondrial responses, assimilate translocation, water and nutrient intake and transportation, and many more^19^. Given the benefits of green synthesized nTiO_2_, including its sustainable benefits, improved bioactivity, reduced toxicity, and environmentally friendly production, this study aimed to better understand how green synthesized nTiO_2_ contributes to the increased resilience of soybean plants to salt stress, providing a new and ecologically friendly method of improving soybean plants.

## Materials and methods

### Preparation of nTiO_2_

For preparation of *Aloe vera* extract, fresh and healthy leaves were gathered and cleaned with tap water and then distilled water to get rid of any contaminants or dirt. After adding 250 g of the leaves to 1000 mL of distilled water, the mixture was heated for two hours at 90 °C. Whatman No. 1 filter paper was used to filter the extract once it had cooled. After filtering, the extract was kept for further research at -4 °C.

**Fig. 1:**
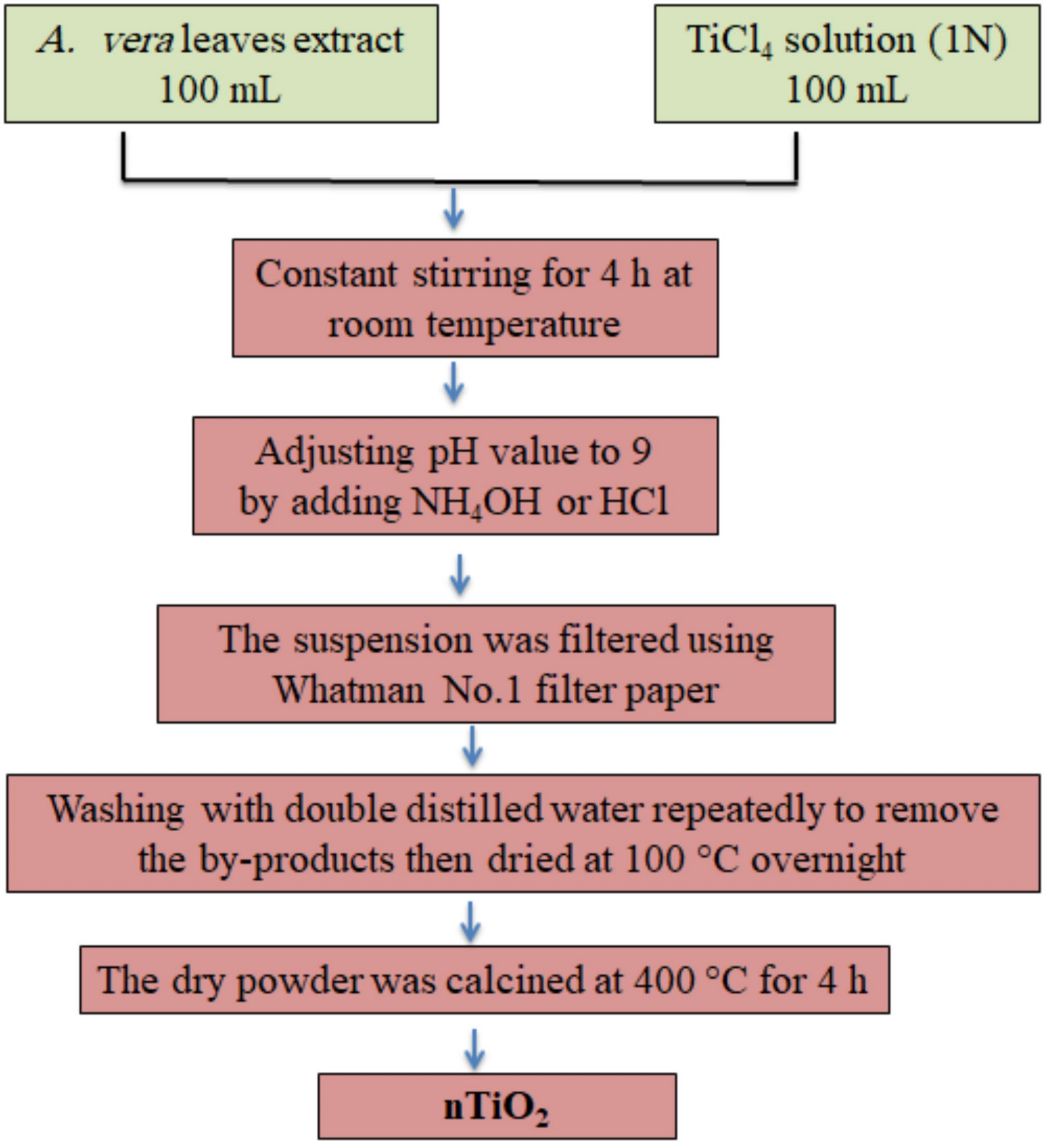
Schematic flowchart for the synthesis of nTiO_2_.

The nTiO_2_ were prepared using *A. vera* leaves extract and titanium tetrachloride as a precursor using the approach of Hanafy et al.,^20^ as shown in Fig. 1. The biosynthesized NPs were characterized using several techniques and recently published by Abdalla et al.,^21^. A suspension of 30 ppm of nTiO_2_ was done using deionized water for further application in soybean plants. This suspension was sonicated in a bath sonicator (Branson’s Model B200 ultrasonic) for 4 h.

### Soil, plant culture, treatments and sampling

Soil was collected from Sharqia Governorate and the main chemical and physical characteristics were ascertained in a 1:5 w/v soil-water extract^22,23^. The soil had a clay texture, a pH of 7.64, and an electric conductivity of 1.15 dS/m, saturation percent of 54.57%, anions content of SO_4_ ^− 2^ = 3.18, Cl^−^ = 2.1, (HCO_3_)^−^ = 0.37, (CO_3_)^−^ = 0.05 meq/100 g soil and cations content of Na = 0.8, K = 0.35, Mg = 1.45, and Ca = 2.19 meq/100 g soil. Pot experiment with soybean (*Glycine max* L. Giza 35) was carried out in the greenhouse of the Botany and Microbiology Department, Faculty of Science, Zagazig University. About 2 kg of soil was taken in plastic pot of 24 cm diameter and irrigated with tap water. In the following day, 10 seeds were sown in each pot. The plants were cultivated in a greenhouse with a 12-hour light/dark cycle (light period at 25 ± 2ºC, dark period at 20 ± 2ºC). Young seedlings were only irrigated with fresh water during the first few weeks of the plantation. To reach the proper density, five plants were then maintained in every pot.

The experiment was done using six different concentrations of NaCl (0, 25, 50, 100, 150 and 200 mM) and two nTiO_2_ concentrations (0 and 30 ppm) with a total 12 treatments, and each treatment was 3 replicas (36 pots). The pots were arranged randomly within the greenhouse to avoid positional bias and irrigation volume was standardized across all treatments to maintain uniform soil moisture levels. After two weeks from sowing, NaCl solutions were added gradually and irrigations were performed at sunset, two times a week by different salt concentrations. In a regular with salt irrigation, a constant volume (5 ml/plant) of nTiO_2_ at 30 ppm concentration was sprayed twice a week by a hand pump sprayer. Non treated plants were used as control irrigated and sprayed with water.

**Fig. 2:**
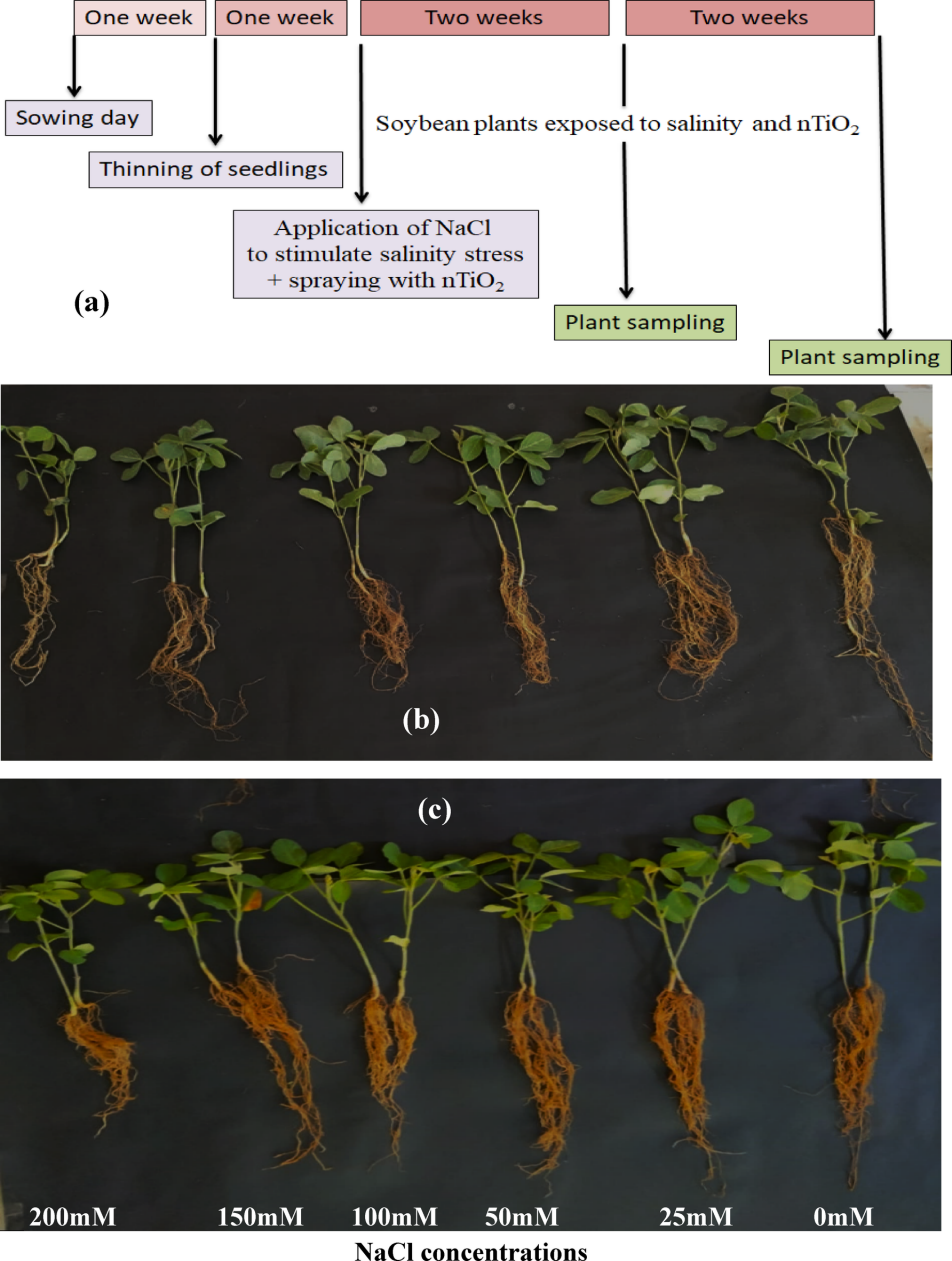
(**a**) Experimental setup overview, (**b**) 15 days-old soybean plants treated with different concentrations of NaCl without nTiO_2_ spraying and (**c**) 15 days-old soybean plants treated with different concentrations of NaCl with nTiO_2_ spraying.

At intervals of 2 weeks, both control and treated plant samples were collected at fixed time in 2 periods (15 and 30 days after treatment with salt). The experimental overview was shown in Fig. 2a. Part of these samples is used for measuring phenotypic variations, leaf water content and pigment fractions; another part was frozen in liquid nitrogen and stored at -20^o^C for biochemical analysis (lipid peroxidation and non-enzymatic antioxidants). As well, some samples were dried in an electrical oven for 3 days at 60^o^C for measuring the dry weight (dwt), carbohydrates and the elemental analysis (Na^+^, K^+^ and Mg^++^).

#### Measurement of phenotypic variations, leaf water content and pigment fractions

Total fresh weight (Tfwt, g) and total dry weight (Tdwt, g) of soybean were measured and number of leaves was counted after 15 and 30 days from salt application. To test the aforementioned characteristics, three plants were randomly selected from each treatment.

The method outlined by Barr and Weatherley^24^ was used to measure the water content (WC), relative water content (RWC), and water saturation deficit (WSD). After cutting the leaf into 5–10 cm^2^, it was immediately weighed to determine its fwt. The leaf sample was left in a Petri dish with deionized water for four hours at room temperature. The sample was removed from the water after four hours, and the surface water was then collected and weighed one more to determine the completely turgid weight (twt). The sample was dried for 24 h at 60 °C in an oven before being weighed once more (dwt). The following formulas were utilized to determine WC, RWC, and WSD:

$$\:\text{R}\text{W}\text{C}\:=\frac{\:(\:\text{f}\text{w}\text{t}\:\--\:\text{d}\text{w}\text{t})\:}{(\text{t}\text{w}\text{t}\--\:\text{d}\text{w}\text{t})}\times\:100$$

$$\:\text{W}\text{C}\:=\:\frac{\text{f}\text{w}\text{t}\--\:\text{d}\text{w}\text{t}\:}{\text{f}\text{w}\text{t}}\times\:100$$

$${\text{WSD}}\,=\,{\text{1}}00--{\text{ RWC}}$$

The Metzner et al.^25^ method was used to determine the pigment content. Using pre-washed sand and 5 mL of an 85% cold aqueous acetone solution, fresh soybean leaves (0.125 g) were pulverised in a mortar. After centrifuging the homogenate, the supernatant was diluted with 85% acetone to a predetermined volume (10 mL). Using a spectrophotometer, the optical density was stated at 663, 644, and 452.5 nm in relation to a blank of 85% acetone. The pigment content of the samples was determined using the following formulas and expressed in µg/mL.

$$\begin{gathered} {\text{Chlorophyll a }}\left( {{\text{Chl}}.{\text{ a}}} \right)\,=\,{\text{1}}0.{\text{3 }}{{\text{A}}_{{\text{663}}}}--0.{\text{918 }}{{\text{A}}_{{\text{644}}}}. \hfill \\ {\text{Chlorophyll b }}\left( {{\text{Chl}}.{\text{ b}}} \right)\,=\,{\text{19}}.{\text{7 }}{{\text{A}}_{{\text{644}}}}--{\text{ 3}}.{\text{87}}0{{\text{A}}_{{\text{663}}}}. \hfill \\ {\text{Carotenoid}}\,=\,{\text{4}}.{\text{2 }}{{\text{A}}_{{\text{452}}.{\text{5}}}} - \left( {0.0{\text{264 Chl}}.{\text{ a}}\,+\,0.{\text{426 Chl}}.{\text{ b}}} \right). \hfill \\ \end{gathered}$$

Note: A = Light absorption in wavelengths 663, 644 and 452.5 nm.

Following calculation, the pigment concentration was reported as mg/g fwt.

#### Determination of the total soluble carbohydrates content and lipid peroxidation

According to Dubois et al.,^26^, the phenol sulphuric acid method was used to measure carbohydrates. Following calculation, the total soluble carbohydrate content was represented as mg/g dwt in the manner described below:

$$\:\:\text{G}\text{l}\text{u}\text{c}\text{o}\text{s}\text{e}\:\left(\frac{\text{m}\text{g}}{\text{g}}\right)\:=\:\frac{\text{m}\text{g}/\text{m}\text{L}\:\times\:\text{e}\text{x}\text{t}\text{r}\text{a}\text{c}\text{t}\:\text{v}\text{o}\text{l}\text{u}\text{m}\text{e}\:\times\:\text{d}\text{i}\text{l}\text{u}\text{t}\text{i}\text{o}\text{n}\:\text{f}\text{a}\text{c}\text{t}\text{o}\text{r}\:}{\begin{array}{c}dwt\:of\:sample\:\left(\text{g}\right)\times\:1000\end{array}}$$

Malondialdehyde (MDA) concentration, which was calculated by homogenizing known tissue weight with 5 mL of 5% (w/v) trichloroacetic acid and centrifuging at 8000 rpm for 10 min, was used to measure the degree of lipid peroxidation^27^. Then, 0.4 mL of the supernatant was mixed with 0.4 mL of 5% trichloroacetic acid that contained 0.67% (w/v) thiobarbituric acid (TBA). Using a spectrophotometer, the absorbance was measured at 532 and 600 nm. An extinction coefficient of 155 mM^− 1^ cm^− 1^ was used to quantify MDA, and the result was represented as nmol/g fwt using the subsequent formula:

$${\text{MDA }}\left( {{\text{nmol}}/{\text{g fwt}}} \right)=[({{\text{A}}_{{\text{532}}}} - {{\text{A}}_{{\text{6}}00}}) \times {\text{ V }} \times {\text{ 1}}000/\varepsilon ] \times {\text{ wt}}$$

where $\varepsilon$ is the specific extinction coefficient (= 155 mM^− 1^ cm^− 1^), V is the volume of the extract, wt is the weight of the leaf, A is the absorbance.

#### Determination of non-enzymatic antioxidants (proline; pro, total phenolic content; TPC and total flavonoid content; TFC)

Pro content was calculated using the Bates et al.,^28^ method. In 10 mL of 3% aqueous sulphosalicylic acid, 0.5 g of soybean tissue were homogenized. Two mL of glacial acetic acid and two mL of acid ninhydrin were combined with two ml of the filtrate in a glass test tube, which was then heated for one hour in a boiling water bath. The tubes were submerged in an ice bath to halt the reaction. After adding four mL of toluene to the reaction mixture, it was well agitated for 15–20 s. Then, organic and inorganic phases are separated, obtaining the chromophore dissolved in toluene. At 520 nm, a spectrophotometer was used to quantify the optical density of the generated color. The formula for expressing Pro as µg/g fwt was as follows:

$$\:{\upmu\:}\text{g}/\text{g}\:\text{f}\text{w}\text{t}\:=\frac{{\upmu\:}\text{g}\:\text{P}\text{r}\text{o}/\text{m}\text{L}\:\times\:\text{m}\text{L}\:\text{t}\text{o}\text{l}\text{u}\text{e}\text{n}\text{e}\:\times\:5\:}{115.5\:\times\:\:\text{f}\text{w}\text{t}\:\text{o}\text{f}\:\text{s}\text{a}\text{m}\text{p}\text{l}\text{e}\:}$$

Note: − 115.5 is the molecular weight of Pro.

After 95% ethanol extraction, 1 mL of the extract was combined with 1 mL of Folin reagent and 1 mL of 20% Na_2_CO_3_ to determine the TPC in soybean leaf tissues quantitatively^29^. At 650 nm, the absorbance was measured with a spectrophotometer. The TPC was tested using gallic acid as a reference, and the results were reported as mg/g fwt of gallic acid equivalent (GAE).

Following Zou et al.,^30^ technique, TFC was evaluated in soybean plant leaves using the aluminium chloride colorimetric test following extraction with 95% ethanol. Four mL of distilled water were mixed with a 100 µL aliquot of alcoholic extract. 0.3 mL of 5% sodium nitrite was added at zero time. 0.3 mL of 10% aluminium chloride was added after 5 min. Two mL of 1 M sodium hydroxide were added to the liquid after six minutes. A spectrophotometer was used to detect absorbance at 510 nm in comparison to a blank. The quercetin standard curve was used to determine TFC, which was then represented as mg/g fwt of quercetin equivalent (QE).

#### Elemental analysis of sodium (Na^+^), potassium (K^+^) and magnesium (Mg^++^) in soybean shoot and roots

The soybean samples (shoot and root; 0.5 g), after being oven dried at 60 °C, were crushed. Then, they were incinerated in a muffle furnace (Thermolyne 48000 model) at high temperature (525–600 °C) for 3 h. Ash of plant samples were dissolved in 10 mL of 2% HNO_3_ and kept in an incubator overnight at 50 °C. After that, the solutions were filtered and reached total volume (25 mL) by deionized water following the method of Ward and Johnston^31^. Na^+^, K^+^ and Mg^++^ were determined spectrophotometrically using atomic absorption spectrophotometer (AAS; Buck scientific 210VGP) at the Central Laboratory, Faculty of Veterinary medicine, Zagazig University. Na^+^, K^+^ and Mg^++^ were computed using the subsequent formula:

$${\text{Element }}\left( {{\text{ppm}}} \right)\,=\,{\text{R}}*{\text{D}}/{\text{dwt}}$$

where R is the element concentration reading in parts per million (ppm) from the AAS digital scale, D is the prepared sample’s dilution, and dwt is the sample’s dry weight.

### Data and statistical evaluation

The impact of nTiO_2_ and salinity stress on growth, biochemical components, and minerals was determined using one-way analysis of variance (ANOVA). Following post hoc analysis, means were compared using Duncan’s multiple comparison tests (*p* ≤ 0.05) with the SPSS statistical software (SPSS Inc., Chicago, IL, USA). Excel was used to plot the figures. Past was used to evaluate the principal component analysis (PCA) and Pearson’s correlation matrix.

## Results

### Impact of nTiO_2_ on phenotypic variations, leaf water content and chlorophylls of soybean plants exposed to salinity

Data for phenotypic variation e.g. Tfwt, Tdwt and leaves number of soybean are listed in Fig. 2b, c; Table 1. Analysis of variance for all of these characteristics revealed that the treatments (control, salt, nTiO_2_, and their interaction) differed significantly (*p* ≤ 0.05). Salt stress had a major impact on plant growth, but applying nTiO_2_ greatly enhanced it. We found that applying salt solutions significantly decreased these metrics; for example, applying a 50 mM salt solution reduced the soybean’s Tfwt (3.78 g ± 0.10 cd), and Tdwt (0.574 g ± 0.011e) in comparison to the corresponding controls. A salt solution at a higher concentration (150 mM) produced an inhibitory effect and reduced the Tfwt (2.7 g ± 0.071 h) and Tdwt (0.5 g ± 0.006 h) respectively, in contrast to the respective controls. However, noteworthy findings emerged in the nTiO_2_ sprayed plants where nTiO_2_ mostly improved total fresh and dry matter in the salt stressed plants (Fig. 2c). Applying nTiO_2_ alone to the plants, 30 ppm, increased the Tfwt (19.5%) and Tdwt (24.2%), compared to their respective controls. Under 50 mM salt solution, when 30 ppm of plant-based nTiO_2_ was applied, the Tfwt and Tdwt of the plants increased noticeably (24.8 and 21.9%) compared to plants grown under salt only. Similarly, these nTiO_2_ enhanced the Tfwt (14.8%) and Tdwt (20%), at 150 mM of salt solution in contrast to plants that are grown under stress only.

**Table 1:** Total fresh weight (Tfwt), total dry weight (Tdwt) (g) and leaves number of soybean plants grown under different NaCl concentrations and sprayed with or without nTiO_2_.

Treatments		Tfwt (g)		Tdwt (g)		Leaves number	
NaCl (mM)	NPs (30 ppm)	Days after salt treatment					
		15	30	15	30	15	30
0	− nTiO_2_	3.5 ± 0.01bc	4.2 ± 0.11ab	0.52 ± 0.013b	0.62 ± 0.02c	15 ± 0.396b	17 ± 0.449b
	+ nTiO_2_	4.2 ± 0.11a	5.02 ± 0.13a	0.59 ± 0.01a	0.77 ± 0.02a	18 ± 0.476a	20 ± 0.529a
25	− nTiO_2_	3.41 ± 0.091bc	3.99 ± 0.105bc	0.44 ± 0.01c	0.657 ± 0.017bc	15 ± 0.396b	16 ± 0.42bc
	+ nTiO_2_	4.06 ± 0.11a	4.87 ± 0.13c	0.49 ± 0.012b	0.733 ± 0.019a	17 ± 0.449b	19 ± 0.502ab
50	− nTiO_2_	3.2 ± 0.08d	3.78 ± 0.10 cd	0.45 ± 0.011c	0.574 ± 0.011e	14 ± 0.37c	16 ± 0.396c
	+ nTiO_2_	3.7 ± 0.1b	4.72 ± 0.13ab	0.46 ± 0.013b	0.7 ± 0.02bc	15 ± 0.396b	16 ± 0.42bc
100	− nTiO_2_	3.1 ± 0.08d	3.7 ± 0.096f	0.43 ± 0.01c	0.57 ± 0.009f	12 ± 0.317d	13 ± 0.343d
	+ nTiO_2_	3.44 ± 0.091c	4.48 ± 0.118bc	0.41 ± 0.01d	0.6 ± 0.014d	13 ± 0.34 cd	15 ± 0.396d
150	− nTiO_2_	1.9 ± 0.052f	2.7 ± 0.071 h	0.32 ± 0.007e	0.5 ± 0.006 h	10 ± 0.264e	5 ± 0.132f
	+ nTiO_2_	2.7 ± 0.07e	3.1 ± 0.082 g	0.35 ± 0.009d	0.6 ± 0.008 g	10 ± 0.264e	10 ± 0.264e
200	− nTiO_2_	1.5 ± 0.04 g	–	0.17 ± 0.003 g	–	7 ± 0.18f	–
	+ nTiO_2_	1.6 ± 0.04 g	2.3 ± 0.059i	0.29 ± 0.006f	0.5 ± 0.006 h	10 ± 0.26e	10 ± 0.291e

*Values are given as means ± standard errors from three replicates. Statistical comparisons were performed using one-way ANOVA and Duncan multiple range test (*p* < 0·05). Different letters inside the column indicate significant difference between treatments.

Water status (WC, RWC and WSD) of nTiO_2_ sprayed and non-sprayed soybean plants were greatly impacted when salt stress was applied as shown in Table 2. Regarding RWC, results showed that soybean plants sprayed with nTiO_2_ possess the highest RWC when not under salt stress condition (60.04%) after 30 days of salt application. RWC of soybean plants exposed to 100 and 150 mM NaCl decreased by 14.011 and 19.88% compared to control ones grown under non-salt stress condition after 30 days. RWC and WC in nTiO_2_ and non-sprayed plants were markedly lessened with increasing salt stress. Moreover, soybean plants sprayed with nTiO_2_ had significantly higher values than non sprayed ones regardless of salt treatments.

**Table 2:** Water status [relative water content (RWC), water content (WC) and water saturation deficit (WSD)] of soybean plants grown under different NaCl concentrations and sprayed with or without nTiO_2_.

Treatments		RWC%		WC%		WSD%	
NaCl (mM)	NPs (30 ppm)	Days after salt treatment					
		15	30	15	30	15	30
0	− nTiO_2_	40.67 ± 1.07f	50.6 ± 1.3 cd	58.3 ± 1.5c	76 ± 2.01f	59.32 ± 1.5e	49.33 ± 1.3de
	+ nTiO_2_	57.67 ± 1.53a	60.04 ± 1.58a	66.14 ± 1.78a	93.9 ± 2.49a	42.33 ± 1.12i	39.96 ± 1.06 g
25	− nTiO_2_	38.3 ± 1.01 g	48.5 ± 1.28e	55.4 ± 2.31e	74.65 ± 2.610 g	61.67 ± 1.63d	51.5 ± 1.4d
	+ nTiO_2_	55.02 ± 1.45b	57.26 ± 1.51b	63.2 ± 1.88b	91.3 ± 2.41b	44.9 ± 1.19 h	42.7 ± 1.13f
50	− nTiO_2_	36.91 ± 0.98gh	45.17 ± 1.19f	49.6 ± 2.08ef	70.2 ± 2.54gh	63.09 ± 1.66 cd	54.84 ± 1.45bc
	+ nTiO_2_	50.76 ± 1.3c	55.63 ± 1.47c	59.56 ± 1.9c	86.14 ± 2.3c	49.24 ± 1.30 fg	44.37 ± 1.17e
100	− nTiO_2_	32.072 ± 0.8 h	43.51 ± 1.15 g	45.2 ± 2.09gh	66.03 ± 2.38 h	67.93 ± 1.7c	56.49 ± 1.49b
	+ nTiO_2_	48.96 ± 1.29d	51.09 ± 1.35d	57 ± 1.746d	84.22 ± 2.3d	51.04 ± 1.3 g	48.90 ± 1.3de
150	− nTiO_2_	29.8 ± 0.788i	40.54 ± 1.07gh	31.5 ± 1.719 h	50.1 ± 1.32i	70.20 ± 1.86b	59.45 ± 1.572a
	+ nTiO_2_	44.5 ± 1.177e	49.89 ± 1.32d	55.3 ± 2.01e	80.28 ± 2.12e	55.5 ± 1.47f	50.12 ± 1.3d
200	− nTiO_2_	23.3 ± 0.62j	–	29.48 ± 1.39i	–	76.67 ± 2.028a	–
	+ nTiO_2_	40.7 ± 1.07f	45.6 ± 1.2de	46 ± 2.01 g	78.69 ± 2.081ef	59.93 ± 1.6e	54.39 ± 1.43c

*Values are given as means ± standard errors from three replicates. Statistical comparisons were performed using one-way ANOVA and Duncan multiple range test (*p* < 0·05). Different letters inside the column indicate significant difference between treatments.

One of the most significant elements that impact a plant’s ability to grow and develop is the level of chlorophyll in it. The effects of salinity, nTiO_2_ and their interactions on the recorded photosynthetic pigments (chlorophylls a, b and carotenoids) of the soybean plant leaves grown throughout the growth period are presented in Table 3. Data showed that chlorophylls and carotenoids in the leaves were decreased significantly under salt stress, whereas the foliar application of nTiO_2_ showed a less decrease where, the interaction treatments showed that nTiO_2_ lessened the harmful effect of salinity throughout the experimental period.

**Table 3:** Pigments content (mg/g fwt) of soybean plants grown under different NaCl concentrations and sprayed with or without nTiO_2_.

Treatments		Chlorophyll a		Chlorophyll b		Carotenoids	
NaCl (mM)	NPs (30 ppm)	Days after salt treatment					
		15	30	15	30	15	30
0	− nTiO_2_	1.8 ± 0.05ab	2.04 ± .01b	0.74 ± 0.02ab	0.8 ± 0.01b	0.69 ± 0.032b	0.74 ± 0.02b
	+ nTiO_2_	2.01 ± 0.051a	2.2 ± .009a	0.9 ± 0.022a	1.06 ± 0.02a	0.99 ± 0.03a	1.08 ± 0.02a
25	− nTiO_2_	1.704 ± 0.033d	1.3 ± 0.009 cd	0.772 ± 0.012b	0.45 ± 0.015ab	0.61 ± 0.024c	0.65 ± 0.02c
	+ nTiO_2_	1.75 ± 0.038b	1.51 ± 0.01c	0.8 ± 0.014ab	0.64 ± 0.012ab	0.965 ± 0.025ab	1.02 ± 0.02a
50	− nTiO_2_	1.373 ± 0.033f	1.23 ± 0.007d	0.72 ± 0.01c	0.401 ± 0.009e	0.537 ± 0.019d	0.62 ± 0.01c
	+ nTiO_2_	1.57 ± 0.04c	1.25 ± 0.009 cd	0.782 ± 0.011b	0.53 ± 0.008c	0.92 ± 0.02ab	1.01 ± 0.015a
100	− nTiO_2_	1.093 ± 0.033 g	1.0 ± 0.006e	0.71 ± 0.004d	0.35 ± 0.005f	0.48 ± 0.015e	0.6 ± 0.013c
	+ nTiO_2_	1.32 ± 0.034de	1.12 ± 0.003d	0.79 ± 0.011bc	0.49 ± 0.004d	0.69 ± 0.019b	0.81 ± 0.013b
150	− nTiO_2_	0.966 ± 0.012 h	0.92 ± 0.005f	0.664 ± 0.011e	0.31 ± 0.008 fg	0.4 ± 0.014 g	0.517 ± 0.009d
	+ nTiO_2_	1.223 ± 0.019ef	1.03 ± 0.005e	0.74 ± 0.008 cd	0.44 ± 0.004de	0.68 ± 0.013b	0.62 ± 0.012c
200	− nTiO_2_	0.25 ± 0.006gh	–	0.18 ± 0.006 g	–	0.397 ± 0.006i	–
	+ nTiO_2_	1.08 ± 0.008 fg	0.59 ± 0.01 g	0.39 ± 0.01f	0.18 ± 0.014 g	0.59 ± 0.011 h	0.52 ± 0.021d

*Values are given as means ± standard errors from three replicates. Statistical comparisons were performed using one-way ANOVA and Duncan multiple range test (*p* < 0·05). Different letters inside the column indicate significant difference between treatments.

### Variations of carbohydrate content and lipid peroxidation as a result of N TiO_2_ application in soybean plants exposed to salinity

**Fig. 3:**
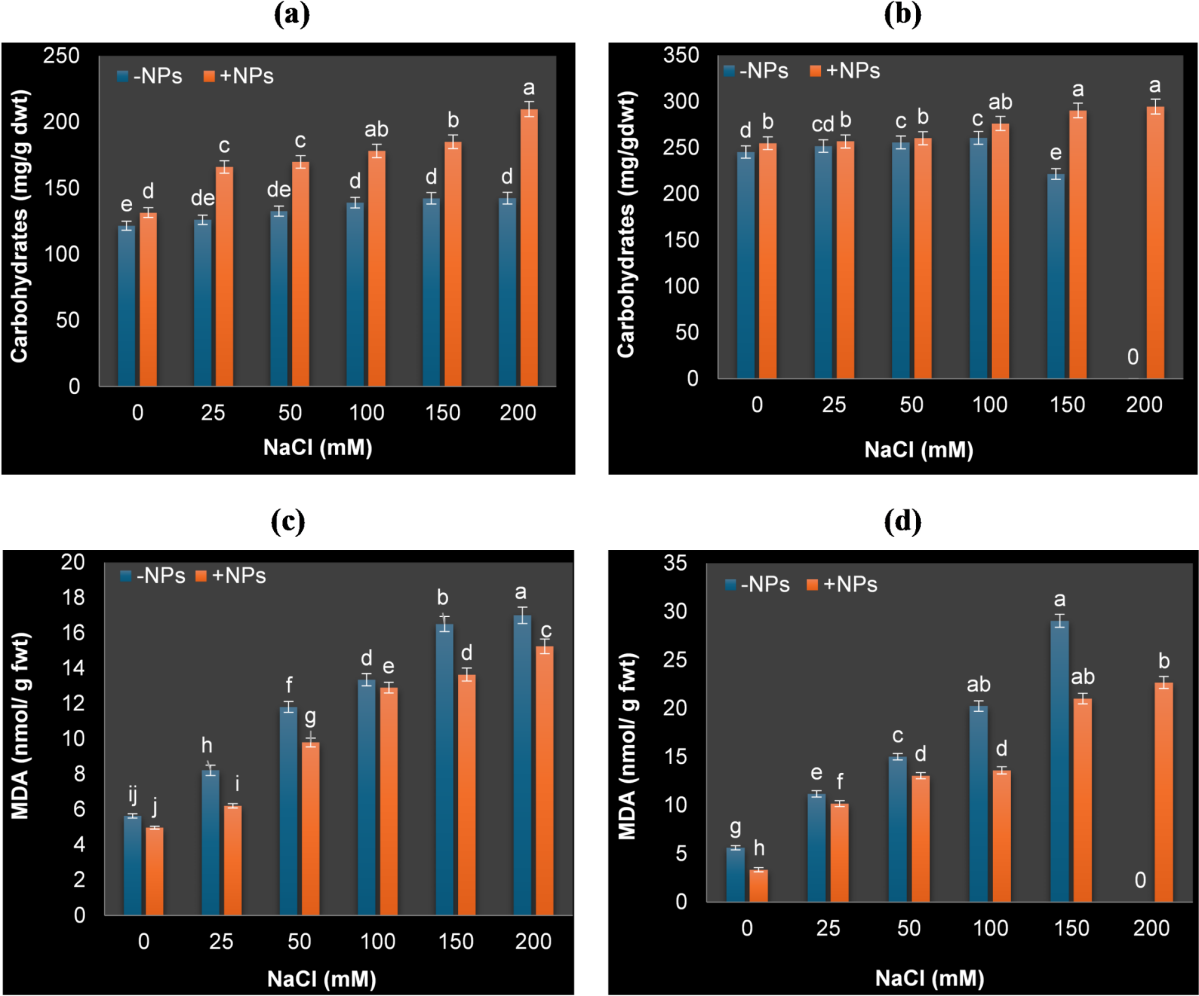
Total soluble carbohydrates (mg/g dwt) and malondialdehyde content (MDA; nmol/g fwt) of soybean plants grown under different NaCl concentrations and sprayed with or without nTiO_2_ after 15 days (**a**,**c**) and 30 days (**b**,**d**) of salt application. *Data represent means ± standard errors (error bars) of three biological replicates. Different letters above columns indicate significant difference (*p* < 0·05), according to a Duncan multiple range test.

Figure 3a showed the effect of different NaCl concentrations, nTiO_2_ application and their interaction on total soluble carbohydrates content. In this investigation, increasing salt concentrations led to a rise in the production of carbohydrates in soybean plants. Also, the exogenous application of nTiO_2_ significantly caused further increase of carbohydrates content under salt stress. It was apparent that salinity stress increased lipid peroxidation of membranes and subsequently increased MDA content of soybean plant leaves. With increasing salt concentrations, there was a subsequent increase in MDA content, recording higher MDA values at higher salt concentration after 30 days of application. In nTiO_2_ sprayed soybean plants, MDA content was significantly decreased under control and salt stressed conditions compared to salt stressed plants only. Furthermore, results in Fig. 3b revealed that the increasing in MDA content was reached to 260.32 and 417.08% at 100 and 150 mM NaCl, respectively in non-nTiO_2_ plants after 30 days while with nTiO_2_ application, these percentages were decreased (142.17 and 274.19) in comparison with plants grown under the control condition. Of particular note, the inhibitory effect of salinity on lipid peroxidation of soybean plants was somewhat lessened by using nTiO_2_.

### Variations in non-enzymatic antioxidants (Pro, TPC and TFC)

**Fig. 4:**
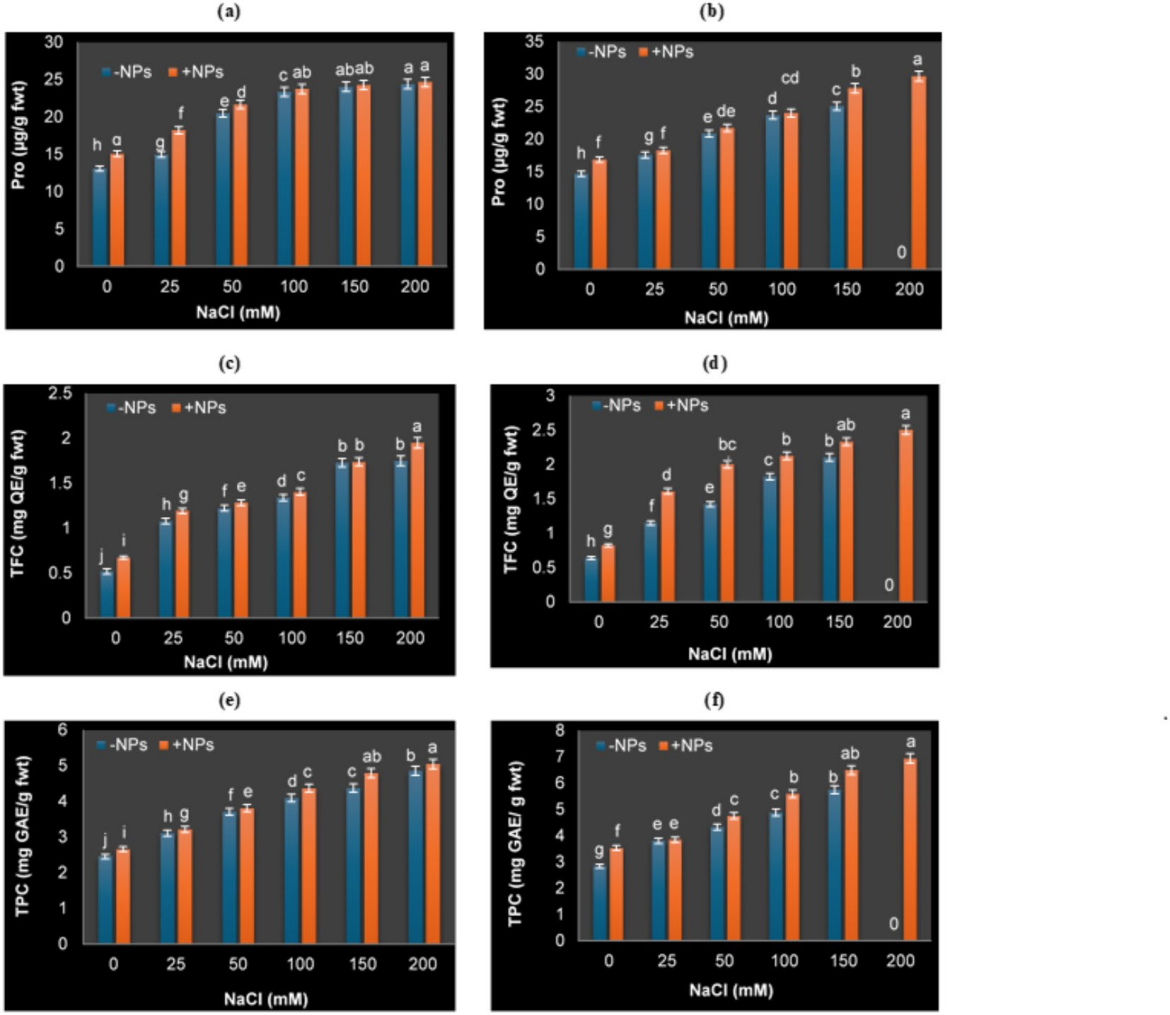
Proline content (Pro; µg/g fwt), total flavonoid content (TFC; mg QE/g fwt) and total phenolic content (TPC; mg GAE/g fwt) of soybean plants grown under different NaCl concentrations and sprayed with or without nTiO_2_ after 15 days (**a**,**c**,**e**) and 30 days (**b**,**d**,**f**) of salt application. Data represent means ± standard errors (error bars) of three biological replicates. Different letters above columns indicate significant difference (*p* < 0·05), according to a Duncan multiple range test.

Results in Fig. 4a, b demonstrated that, in comparison to the control, salt stress caused a noticeable rise in the Pro content in the leaves of both nTiO_2_ and non-nTiO_2_ sprayed soybean plants, where the maximum level was seen at 150 and 200 mM NaCl. Furthermore, Pro content in leaves of nTiO_2_ applied soybean plants was higher than that in non-nTiO_2_ applied plants grown under control or salt stressed condition. Where the Pro content in non-nTiO_2_ soybean plant leaves grown at 50 mM NaCl were 20.49 and 20.87 µg/g fwt, while with nTiO_2_ application, this value was increased (22.67 and 23.74 µg/g fwt) after 15 and 30 days, respectively.

The production of secondary metabolites, including flavonoids and phenolic compounds, is increased under salinity stress (Fig. 4b-e). To determine whether a non-enzymatic mechanism driving nTiO_2_’ induction of salt tolerance in soybean plants, TPC and TFC were detected after 15 days and 30 days (Fig. 4b-e). As shown in this figure, the TPC and TFC of soybean plants were measured as 2.46 mg GAE/g fwt and 0.52 mg QE/ g fwt in the control. As well, noticeable and gradual increases in their contents were noticed with increasing NaCl concentrations. The TPC increased by 52.37% and 71.88% in plants challenged with 50 and 100 mM NaCl, respectively; these percentages were increased by 67.31% and 96.44% with green produced nTiO_2_ treatment compared to control in untreated plants only after 30 days.

### Changes of Na^+^ K^+^, and Mg^++^ in shoots and roots of soybean

Application of nTiO_2_, salt stress and their interactions significantly affected nutrients content of soybean plants. The present study showed that salt stress caused a notable rise in Na^+^ and a reduction in K^+^ and Mg^++^ buildup in leaves and roots of soybean as shown in Tables 4 and 5. However, interestingly, nTiO_2_ treated plant exhibited reduced Na^+^ accumulation and enhanced K^+^ and Mg^++^ under NaCl stress. It has been noted that the salt stress led to low K^+^/Na^+^ ratio (high Na^+^/K^+^), while nTiO_2_ application raised this ratio (K^+^/Na^+^).

**Table 4:** K^+^, Na^+^ and Mg^++^ content (mg/0.1 g dwt) in shoot of soybean plants grown under different NaCl concentrations and sprayed with or without nTiO_2_.

Treatments		K^+^		Na^+^		Mg^++^	
NaCl (mM)	NPs (30 ppm)	Days after salt treatment					
		15	30	15	30	15	30
0	− nTiO_2_	2.35±0.06bc	2.5±0.06a	0.003±0.00008 g	0.0031±0.00009f	0.79±0.021b	0.78±0.02b
	+ nTiO_2_	2.39±0.063a	2.49±0.07a	0.0028±0.00007 h	0.003±0.00008f	0.86±0.022a	0.82±0.021a
25	− nTiO_2_	2.306±0.06ab	1.84±0.05c	0.0031±0.00008 g	0.006±0.00017e	0.70±0.018c	0.69±0.0181bc
	+ nTiO_2_	2.308±0.06b	1.95±0.05b	0.003±0.00008 g	0.0058±0.00016e	0.73±0.02c	0.72±0.019ab
50	− nTiO_2_	2.27±0.06c	1.67±0.04d	0.005±0.00012e	0.009±0.0002ab	0.47±0.0071e	0.55±0.0145de
	+ nTiO_2_	2.29±0.06abc	1.83±0.034e	0.004±0.00008f	0.0085±0.00019d	0.54±0.0089d	0.65±0.0171c
100	− nTiO_2_	2.109±0.06 cd	1.49±0.04de	0.006±0.00014d	0.0095±0.0002c	0.45±0.0067e	0.54±0.014f
	+ nTiO_2_	2.19±0.06 cd	1.52±0.02f	0.005±0.00012e	0.0088±0.0002d	0.46±0.007ede	0.58±0.015d
150	− nTiO_2_	1.86±0.05f	1.25±0.03e	0.007±0.00018c	0.012±0.00021b	0.449±0.006f	0.535±0.0035 fg
	+ nTiO_2_	2.09±0.055e	1.36±0.02 g	0.006±0.00015d	0.009±0.0002ab	0.45±0.0066f	0.57±0.0045f
200	− nTiO_2_	1.52±0.04f	–	0.009±0.00024a	–	0.33±0.0059 g	–
	+ nTiO_2_	1.58±0.041 g	1.01±0.064 h	0.008±0.00021b	0.019±0.00025a	0.34±0.006eg	0.43±0.0034 g

*Values are given as means ± standard errors from three replicates. Statistical comparisons were performed using one-way ANOVA and Duncan multiple range test (*p* < 0·05). Different letters inside the column indicate significant difference between treatments.

**Table 5:** K^+^, Na^+^ and Mg^++^ content (mg/0.1 g dwt) in root of soybean plants grown under different NaCl concentrations and sprayed with or without nTiO_2_.

Treatments		K^+^		Na^+^		Mg^++^	
NaCl (mM)	NPs (30 ppm)	Days after salt treatment					
		15	30	15	30	15	30
0	− nTiO_2_	1.67 ± 0.044b	1.71 ± 0.045b	0.09 ± 0.004e	0.12 ± 0.0047f	0.66 ± 0.017b	0.68 ± 0.017b
	+ nTiO_2_	1.71 ± 0.045a	1.78 ± 0.046a	0.084 ± 0.0045e	0.10 ± 0.005 g	0.67 ± 0.017a	0.71 ± 0.018a
25	− nTiO_2_	1.62 ± 0.042c	1.45 ± 0.038	0.11 ± 0.0046d	0.14 ± 0.0049ef	0.524 ± 0.013ab	0.61 ± 0.016c
	+ nTiO_2_	1.64 ± 0.043ab	1.53 ± 0.040c	0.1 ± 0.005d	0.12 ± 0.0051f	0.55 ± 0.0145ab	0.41 ± 0.003d
50	− nTiO_2_	1.409 ± 0.037e	1.353 ± 0.035d	0.15 ± 0.0051 cd	0.18 ± 0.0052d	0.49 ± 0.012d	0.41 ± 0.002de
	+ nTiO_2_	1.54 ± 0.040d	1.515 ± 0.040 cd	0.13 ± 0.0051d	0.15 ± 0.0059e	0.51 ± 0.013c	0.412 ± 0.0029d
100	− nTiO_2_	1.17 ± 0.030d	1.09 ± 0.028de	0.205 ± 0.0054c	0.24 ± 0.006b	0.473 ± 0.012de	0.406 ± 0.0028de
	+ nTiO_2_	1.409 ± 0.037e	1.105 ± 0.029e	0.2 ± 0.0052c	0.21 ± 0.0059c	0.478 ± 0.012de	0.41 ± 0.0028d
150	− nTiO_2_	1.075 ± 0.03dg	0.93 ± 0.024f	0.22 ± 0.0064b	0.27 ± 0.006a	0.459 ± 0.012 fg	0.395 ± 0.002e
	+ nTiO_2_	1.36 ± 0.035f	1.006 ± 0.026ef	0.21 ± 0.006c	0.23 ± 0.006bc	0.46 ± 0.012f	0.40 ± 0.0027de
200	− nTiO_2_	0.75 ± 0.019i	–	0.24 ± 0.0066a	–	0.42 ± 0.010 g	–
	+ nTiO_2_	1.03 ± 0.027gh	0.88 ± 0.023 g	0.23 ± 0.007ab	0.25 ± 0.0076ab	0.451 ± 0.012 fg	0.381 ± 0.0021e

*Values are given as means ± standard errors from three replicates. Statistical comparisons were performed using one-way ANOVA and Duncan multiple range test (*p* < 0·05). Different letters inside the column indicate significant difference between treatments.

### Principal component analysis and correlation matrix among phenotypic, biochemical parameters and minerals

**Fig. 5:**
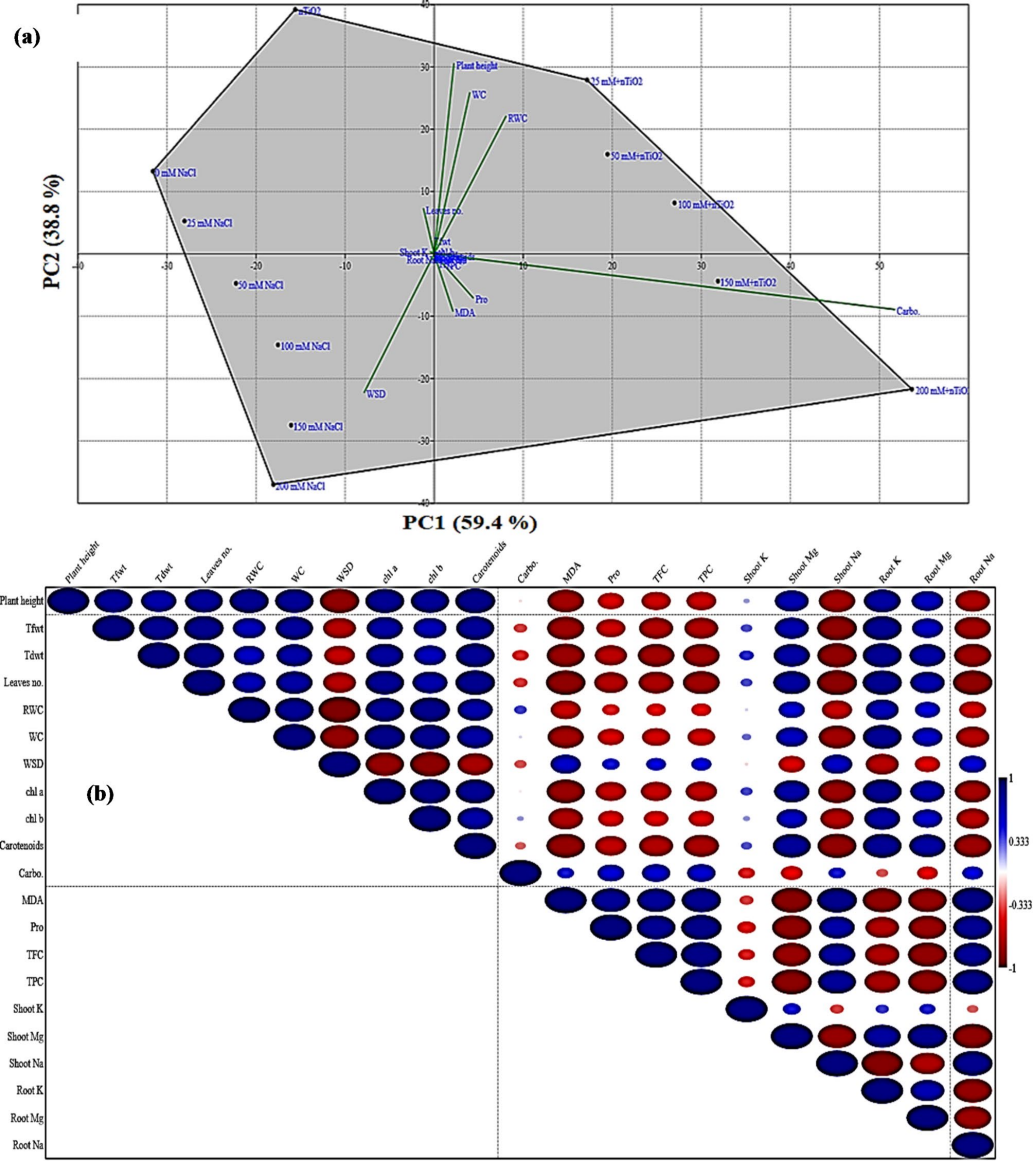
Multivariate analysis: (**a**) Principal component analysis (biplot) between the different treatments and the studied parameters of soybean plants under the effects of salinity and nTiO_2_. (**b**) Pearson’s correlation matrix of 21 traits in soybean plants. **Note**: The intensity of color ranges from blue (positive) to red (negative) and the size of the circles show strength of significant correlation (*p* ≤ 0.05).

**Fig. 6:**
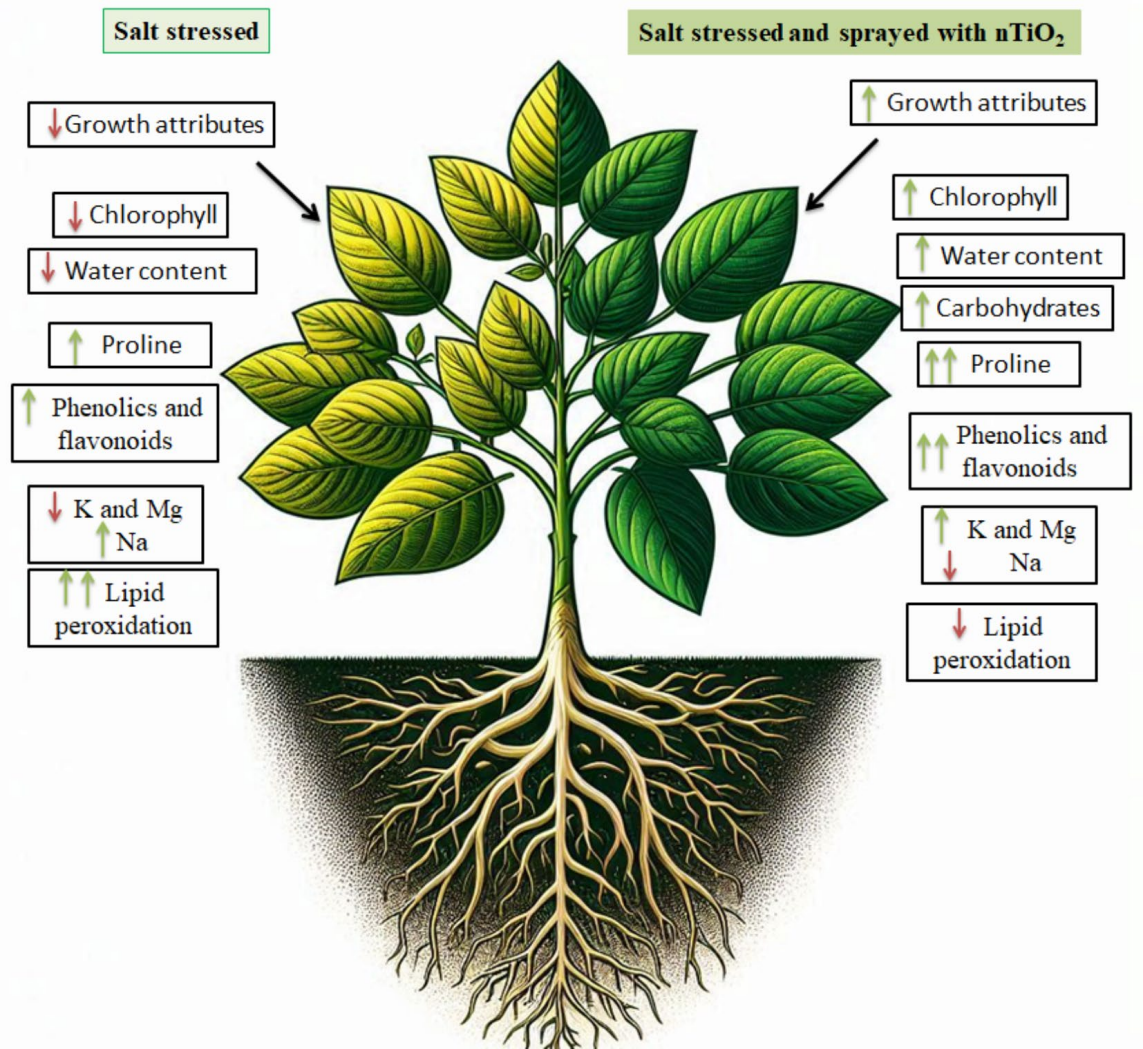
Representation for the effects of salinity and nTiO_2_ on soybean plants (this picture was generated by AI).

Principal component analysis (PCA) shows the association among the phenotypic, physio-biochemical parameters and minerals of soybean plants exposed to the 12 treatments (Fig. 5a). The first principal component (PC1) accounted for 59.4% of the variance in the obtained data, whereas the second component (PC2) captured 38.8% of the variance under the different treatments explained a total of 98.2% overall data variability. The biplot of various parameters indicated that plant height, WC, RWC, and Tfwt were positively correlated with each other and negatively with MDA content. Also, this biplot (Fig. 5a) authenticated the grouping of 12 treatments used in this study. The nTiO_2_ was adjudged as the best treatment and its effect was followed by that of control treatment. Both these treatments were clustered on the upper left-hand rectangles of the plot. The NaCl (50, 100, 150 and 200 mM) treatments posed negative impacts on soybean plants, and this was confirmed by the plot. On the PCA-linked biplot, the most obvious result is that all the salinity treatments were highly associated with WSD and MDA. According to the correlation matrix (Fig. 5b), WSD, MDA, Na content in shoot and roots were negatively correlated with all growth parameters and pigment fractions. Also, this correlation revealed a strong positive correlation between different growth parameters measured (plant height, Tfwt and Tdwt) with all the pigment fractions (chlorophylls a, b and carotenoids) which indicate the role of increasing pigments (increasing photosynthesis) in enhancing growth parameters. Collectively, Fig. 6 summarized the effect of salinity and nTiO_2_ on soybean plants based on the different parameters measured in this study.

## Discussion

The nTiO_2_ were green synthesized using *A. vera* extract and their characterization showed a well-crystallized anatase profile with tetragonal structure of the particles and their sizes ranged between 10 and 25 nm as previously mentioned in our recently studies^21,32^. Employing NPs is seen to be a viable way to get around the challenges associated with plant growth and development under different stresses^33^. Assessment of the impact of nTiO_2_ on soybean plants and research on the related alterations in treated plants was recently done^32^ while further understand the mechanism of resilience is still required.

The present results indicated the inhibition of the measured growth parameters by NaCl treatments. These findings are congruent with the results of salinity effects on wheat, tomato and faba bean plants^19,34,35^ respectively, implying that it might be an unintended result of Na^+^ ions. Salinity stress affects a plant’s growth and its physiochemical properties as well^36^. A notable decrease in growth and biomass was brought on by salt stress because of the increased translocation of Na^+^ from the roots to the shoots or the lack of nutrients^10^. Conversely, application of nTiO_2_ improved the growth parameters and this agreed with Jaberzadeh et al.,^37^ who reported that nTiO_2_ enhanced the growth and yield-related characteristics of wheat plants under stress. In their 2018 study, Rafique et al.^38^ demonstrated that following 60 days of exposure to soil-applied nTiO_2_, *Triticum aestivum* L. plants’ root and shoot lengths and P uptake were significantly (*p* < 0.05) higher between 20 and 60 mg kg^− 1^ than the control (0 mg kg^− 1^ nTiO_2_), but then decreased at 80 and 100 mg kg^− 1^ in comparison to 60 mg kg^− 1^ nTiO_2_. Likewise, the research carried out by Badshah et al.,^16^ demonstrated that the application of nTiO_2_ improved plant development, which may be connected to the synthesis of antioxidant enzymes and osmotic adjustments in plants. In agriculture, nanotechnology is important and aids in crop management. The more acceptable approach is TiO_2_ biosynthesis since it promotes growth and has a notable impact on plant growth when exposed to salinity stress^39^. Applying nTiO_2_ enhanced the absorption of macro- and micronutrients, boosted the growth characteristics of plants (e.g., length, fresh weight, and number of leaves), and lessened the adverse effects of salinity, such as interfering with photosynthesis and essential element absorption^18^.

Plants cultivated under salt stress are exposed to physiological drought because Na^+^ and Cl^−^ ions bind water that the plants require to mobilize^40,41^. The water status of jute and fenugreek plants is impacted by salt stress, which is consistent with findings by Chaudhuri and Choudhuri^42^ and Metwally and Abdelhameed^43^. The buildup of Na^+^ in salty soils causes a drop in osmotic potential during salinity stress, which might lower the soil’s WC^44^. Under conditions of salt stress, abscisic acid is generated, which closes the stomata and lowers the water absorbed by the roots and affects the transpiration process, reducing the WC in the cell^45^. These findings are also agreed with Özdemir et al.,^46^ where they found that specific physiological reactions, such as stomatal closure, increased rates of leaf senescence, and reduced plant growth, happen when plants endure salt stress. When plants are stressed by salinity, they absorb less water overall. Increased osmotic stress from high salt concentrations in soil solution restricts the plant’s ability to absorb water, which impacts leaf WC, stomatal conductance, growth (accelerating senescence and death), and photosynthesis (decrease in chlorophyll concentrations), all of which contribute to a decrease in plant growth^8^.

According to Mahmoud et al.,^47^, in many plant species, NPs can alleviate osmotic stress caused by salt by improving water status and water use efficiency. In this connection, the role of nTiO_2_ in enhancing the performance of soybean plant leaves which represented by WC and RWC, was greatly apparent after 30 days of salt application. Our results are consistent with the ones obtained by^18^, who applied nTiO_2_ to wheat plants impacted by salt and reported comparable results. It has also been demonstrated that nTiO_2_ treatment raises the RWC in stevia in addition to maintaining the cells’ water status^14^. Moreover, plants treated with NPs continue to exhibit increased whole-plant hydraulic conductance, leaf and root water content, stomatal conductance, and transpiration rate^48^. NPs have been shown to enhance root hydraulic conductance by upregulating the expression of the plasma-membrane intrinsic protein aquaporins. This may help to boost water uptake and decrease membrane damage and oxidative stress. According to a study by Elhefnawy et al.^49^, tomato seeds treated with NPs had a moisture content that was 19% higher than that of untreated seeds. These results implied that the NPs encourage water absorption and retention.

Regarding the content of chlorophylls and carotenoids, salt stress decreased them significantly whereas the foliar application of nTiO_2_ showed a less decrease. When salinity stress occurs, it influences the leaf area, the leaf area becomes short and subsequent chlorophyll decreases. Under salinity stress, numerous plant species have been shown to have decreased photosynthetic activity^50,51^. When under salinity stress, stomatal conductance significantly decreases, lowering CO_2_ concentration and net photosynthetic rate^52,53^. Additionally, El-Shawa et al.,^54^ demonstrated that salt stress resulted in a notable decline in the concentrations of chlorophyll and carotenoids in calendula plants as a result of the inhibition of PSII activity and a decrease in chlorophyll and CO_2_ assimilation in leaves due to the accumulation of toxic ions^55^.

Consistent with the present results of increasing chlorophylls and carotenoids following nTiO_2_ application, a research by Rafique et al.^38^ found that when nTiO_2_ were applied to *T. aestivum* L. plants, the amount of chlorophyll increased by 32.3% at 60 mg kg^− 1^ compared to the control, but decreased by 11.1% at 100 mg kg^− 1^. Obviously, applying nTiO_2_ delayed the chloroplast senescence process^56^ and stimulated Rubilose carboxylase activity, increasing the amount of chlorophyll and the rate of photosynthetic activity^57,58^. The treatments with nTiO_2_ regulate the activity of nitrogen metabolism-related enzymes and improve the process of turning inorganic nitrogen into organic nitrogen, together with the synthesis of chlorophyll and proteins^56,59^. Under salt stress, application of the majority of NPs (Ag, ZnO, TiO_2_, Fe, and Se) increased the amount of chlorophyll^60^. According to Phothi and Theerakarunwong^61^, these findings demonstrated the physiological role of nTiO_2_ in boosting light harvesting, initiating photosynthesis, and stimulating the concentration of proteins and pigments. Under salinity stress, nTiO_2_ increased the carotenoid content of wheat cultivars^15^.

In order to survive in saline environments, plants gather fundamental osmolytes such Pro, protein, and carbohydrates as a vital way to compensate for osmotic stress caused by salinity^62^; these osmolytes are non-toxic at elevated levels^63^. Our results of increasing carbohydrates in soybean plants under salt stress and nTiO_2_ application are in line with those of^15^, who applied nTiO_2_ to plants of *T. aestivum* L. under salt stress and reported comparable outcomes. The nTiO_2_ treatment raised Pro and carbohydrate contents in plants, which can aid soybean plants exposed to salt as osmoprotectants by stabilizing membranes and preventing enzyme denaturation^64^. According to numerous reports, the application of NPs can also improve plant resistance to salinity stress by altering the concentrations of solutes like total soluble sugars and amino acids (like Pro)^48,65,66^. This reduces the osmotic shock caused by NaCl stress because of ion toxicity (Na^+^ and Cl^−^). For instance, Farouk and Al-Amri^67^ found that applying Zn NPs to canola (*Brassica napus* L.) plants under salinity stress reduced the negative effects of salt through ionic control and osmolyte production. In a different study, Mohamed et al.,^68^ showed that treating seeds with Ag NPs prior to sowing enhanced the growth, Pro, and soluble sugars of wheat seedlings under salt stress. Likewise, Metwally and Abdelhameed^4^ and Abdelaziz et al.,^69^ stated that NPs application to pea and eggplant plants stimulated the total soluble carbohydrates and total soluble protein.

Carbohydrates are significant organic solutes that may play a key role in stress reduction and aid in maintaining cell homeostasis, through the action of signal molecules, carbon storage, and osmoprotectants. Carbohydrates like sugars (such as glucose, fructose, and trehalose) and starch have been shown to function as metabolite and nutrient signaling molecules and to be involved in the immune system’s response to a number of stressors^70,71^. Additionally, the high carbohydrate accumulation helps to prevent oxidative harms by scavenging ROS and maintaining protein structure during salt stress. Abobatta and Waleed Fouad^72^ stated that soluble sugars are significant osmolytes that, in glycophytes exposed to salty environments, can account for as much as 50% of the entire osmotic potential.

MDA is a measure of membrane stability or, indirectly, a marker of membrane damage caused by high ROS. The stability of the membrane is disturbed when ROS produced by NaCl interacts with membrane proteins, causing peptide chain fragmentation and proteolysis. ROS can attack the lipid molecules in the membranes, thereby rendering the membranes permeable for electrolytes to leach out. As well, ROS causes membranes’ unsaturated lipid component peroxiding, which causes the membranes to lose their integrity and so contain more MDA^73^. Application of NPs decreased lipid peroxidation in plants under salt stress as reported in several studies. For instance, Sheikhalipour et al.,^14^, Alharby et al.,^19^ and Abdel Latef et al.,^39^ confirmed that application of nTiO_2_ reduced the MDA levels in the saline environment. The reason might be due to increased activities of antioxidants in nTiO_2_ treated plants which reduce lipid peroxidation and scavenge the generation of radicals before they react with the membrane lipids^74^. On the contrary, numerous studies have demonstrated that plants treated with higher concentration of metal ions had higher MDA contents^75,76^ which attributed to the generation of high ROS and oxidative stress from metal ions produced from NPs^77,78^. The discrepancy in outcomes may stem from differences in NPs synthesis, concentration, plant species, and growth conditions.

It has been shown that Pro stabilizes subcellular structures (proteins and membranes), buffers cellular redox potential under stress, and acts as a free radical scavenger and carbon and nitrogen storage sink^79^. It helps in maintaining the water balance of plants so that the stress induced is reduced^80^. It also influences protein turn over and directly regulates stress protective proteins^81^. Our results of Pro accumulation align with those of Mustafa et al.^15^, who reported similar results by applying nTiO_2_ on *T. aestivum* L. plants affected by salinity stress. Pro levels in plants are accelerated by nTiO_2_ treatment, which can benefit soybean plants exposed to salt as osmoprotectants by stabilizing membranes and preventing enzyme denaturation^64^. An increase in Pro makes the cell more stress-tolerant and protects cellular structures and cytosolic enzymes.

Phenolics and flavonoids, which are created under stressful circumstances, are essential to the growth and development of plants and also have a significant nutritional component. They also go by the name of antioxidants since they are crucial in minimizing the harm brought on by oxidative stress^7^. The present result showed an increase in the TPC and TFC in soybean plants under salt stress. Similarly, a noticeable increase in their contents was found at 50 and 100 mM NaCl treated tomato seedlings^16^. Furthermore, the findings of phenolics and flavonoid increase in soybean leaves were consistent with previous research on artichoke (*Cynara scolymus* L.) leaves by Rezazadeh et al.,^82^. This could be explained by the discovery that stress causes an increase in enzymes of the flavonoid biosynthesis pathway, such as chalcone synthase and phenylalanine ammonia-lyase^83^.

The current study demonstrated how applying nTiO_2_ can help reducing the negative effects of salt stress by higher accumulation of TPC and TFC. There is also evidence that these compounds have increased protective effects against salt stress, helping plants maintain ROS levels in harmful environments and promoting quick elimination to maintain a stable metabolism. These results align with Mustafa et al.,^15^ findings. According to earlier research by Lafmejani et al.,^84^, this might be the consequence of increased expression of particular biosynthetic enzymes involved in the synthesis of these components and substrate availability. It is yet unclear exactly how the application of NPs affects plant secondary metabolites. NPs may function as elicitors for secondary metabolite formation in plants by triggering several cellular signal transduction pathways (e.g., mitogen-activated protein kinases, calcium flux, and ROS metabolism), according to recent integrated phytochemical and genomic investigations. Therefore, the observed modifications in the aforementioned pathways may result in changes in the levels of gene expression and the activation of metabolic enzymes, which may influence the synthesis of secondary metabolites^85^.

Plants’ ability to regulate their ion homeostasis is disrupted by salt stress^86^. The current investigation showed that salt stress significantly increased Na^+^ and decreased K^+^ and Mg^++^ accumulation. These findings are consistent with those of Elhindi et al.,^87^, who found that salinity causes *Andrographis paniculata* to accumulate more Na^+^ and accumulate less K^+^ and Mg^++^. Because Na^+^ ions compete with K^+^ for binding sites, elevated Na^+^ always inhibits K^+^ absorption. Moreover, Ghassemi-Golezani and Abdoli^88^ revealed that excessive salinity stress frequently prevents rapeseed (*B. napus* L.) plants from absorbing and distributing vital minerals (Mg^++^ and K^+^), which are necessary for the production of chlorophyll.

However, interestingly, nTiO_2_ treated plant exhibited reduced Na^+^ accumulation and enhanced K^+^ and Mg^++^ under NaCl stress. The heightened build-up of nutrients (K^+^ and Mg^++^) with nTiO_2_ application under control and saline conditions improved the plant growth because they are important components of many metabolically active compounds and play a crucial role in several physiological and biological functions, also improved the plant tolerance by inducing many enzymes associated with nutrients assimilation and antioxidant enzymes. Mg^++^ being the component of chlorophyll helps to enhance the photosynthetic rate. NPs also improve the plant’s capacity to effectively absorb and use water and fertilizers from the soil^89^. According to Gohari et al.,^90^, nTiO_2_ has also been shown to hasten the accumulation of other vital elements in plant shoots, including Fe, Ca, Mn, Zn, B, and K. Mustafa et al.,^15^ claim that by enhancing the uptake of vital nutrients and preventing the uptake of Na^+^ ions, nTiO_2_ improves growth and yield characteristics. It works as a standard that provides a sufficient amount of vital elements to plant roots by utilizing the sequestration process of essential elements by NPs. NPs can improve the solubility and bioavailability of certain nutrients in the soil, leading to increased nutrient absorption by plant roots^91^. According to the findings of Alharby et al.,^19^, the addition of nTiO_2_ raised the amounts of P, K, Fe, and Mn in wheat up to 400 mg/kg. At higher doses of nTiO_2_ (600 mg/kg), the concentrations of these elements declined. Additionally, wheat plants showed improvement in P concentration under nTiO_2_ application up to 60 mg/kg, which progressively decreased as nTiO_2_ concentration increased up to 100 mg/kg^38^, which could be because root exudation mobilized the soil P, increasing plant uptake of it^92^. Moreover, nTiO_2_ might stimulate the release of organic acids from roots, leading to rhizosphere acidification. This acidification can alter nutrient availability and uptake. For instance, a study on maize seedlings treated with nTiO_2_ observed increased exudation of citric, lactic, and fumaric acids, accompanied by a decrease in exudate pH^93^. Such changes in root exudation patterns can influence the solubility and mobility of nutrients like K⁺ and Mg²⁺, facilitating their uptake. Different cultivars, plant species, experimental settings, and competition between nTiO_2_ and mineral elements during plant uptake could all contribute to the diversity in nutrient uptake by plants under nTiO_2_.

**Fig. 7:**
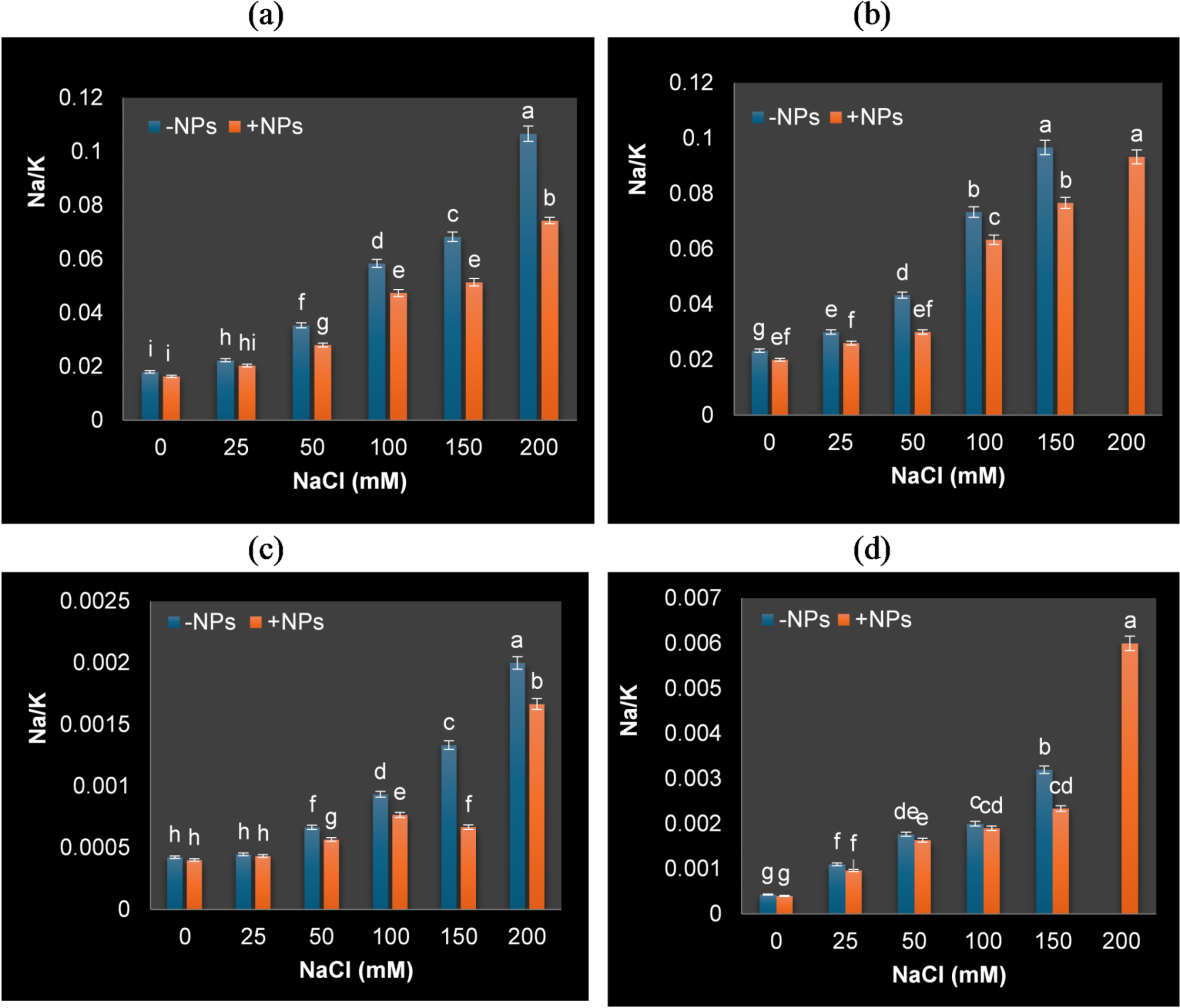
Na^+^/K^+^ in root and shoot of soybean plants grown under different NaCl concentrations and sprayed with or without nTiO_2_ after 15 days (**a**,**c**) and 30 days (**b**,**d**) of salt application. Data represent means ± standard errors (error bars) of three biological replicates. Different letters above columns indicate significant difference (*p* < 0·05), according to a Duncan multiple range test.

It has been noted that the high K^+^/Na^+^ ratio (low Na^+^/K^+^), which is disturbed by salinity stress, is one of the most crucial elements for plant resistance to salinity stress. NPs are known to raise this ratio (Sytar et al., 94; Tahjib-UI-Arif et al., 95), which enhances the plant’s osmotic potential and promotes better plant growth in the face of salinity stress. The current findings (Fig. 7) demonstrated how nTiO_2_ may help plants absorb more K^+^ than Na^+^ when they are under salt stress (low Na^+^/K^+^). Parida and Das^96^ previously discussed the role of K^+^ in plants’ adaptation to salt. They found that plants maintain low concentrations of Na^+^ and high concentrations of K^+^ in the cytosol by controlling the expression and activity of H^+^ pumps, which provide the driving force for transport, as well as K^+^ and Na^+^ transporters.

Maintaining a greater K^+^/Na^+^ ratio is thought to be a key tactic used by plants to lessen harmful alterations brought on by stress^97,98^. Effective removal of harmful ions, such as Na^+^, helps maintain tissue osmotic potential, hence minimizing hyperosmotic consequences. According to the study of Alharby et al.,^19^, enhanced uptake of P, K^+^ and Ca^2+^ due to nTiO_2_ may be as result of selective absorption of these essential ions over deleterious Na^+^ and hence resulting in maintaining lower Na^+^/K^+^ ratio. It appears that the role of the nTiO_2_ in alleviating salt stress is partially due to the prevention of Na^+^ absorption and translocation to shoot tissues.

## Conclusions

This study emphasizes how nTiO_2_ can improve the performance of soybean plants in saline environments. MDA and Na^+^ content in both shoots and roots decreased as a result of the nTiO_2_’s action. Moreover, nTiO_2_ markedly modulated phenotypic attributes, key physiological and biochemical parameters including RWC, Pro, chlorophylls, TPC and TFC. As well, the application of nTiO_2_ enhanced uptake of essential element such as K^+^ and Mg^++^. These effects are involved in bolstering soybean tolerance to the toxic and osmotic stresses induced by salinity. From an applied perspective, these results suggest that nTiO_2_ could serve as a promising nanomaterial for improving soybean cultivation in saline soils, offering a potential strategy to enhance crop productivity in salt-affected agricultural regions. In order to clarify the fundamental mechanisms of NPs interactions in subsequent research, these findings provide the groundwork for a deeper comprehension of NPs impacts. Future studies should focus on validating these findings under field conditions and elucidating the underlying molecular mechanisms.

## Data Availability

Data is provided within the manuscript.

## Abbreviations

nTiO_2_: Titanium dioxide nanoparticles
NPs: Nanoparticles
MDA: Malondialdehyde
ROS: Reactive oxygen species
Pro: Proline
TPC: Total phenolic content
TFC: Total flavonoid content
GAE: Gallic acid equivalent
QE: Quercetin equivalent
Tfwt: Total fresh weight
